# Current Risks and Prevention Strategies Against Vector-Borne Diseases in Cyprus

**DOI:** 10.3390/microorganisms13040726

**Published:** 2025-03-24

**Authors:** Ender Volkan, Panagiotis Karanis

**Affiliations:** Department of Basic and Clinical Sciences, University of Nicosia Medical School, 2414 Nicosia, Cyprus

**Keywords:** infectious diseases, neglected diseases, parasites, malaria, bacteria, viruses, One Health, Cyprus

## Abstract

The island of Cyprus has historically been prone to vector-borne diseases due to its location at the crossroads of three continents. The introduction of novel vectors, microorganisms, or strains in Cyprus, coupled with the global climate change and antimicrobial resistance crisis, can lead to an altered infectious disease landscape and entomological status, causing a rise in vector-borne diseases on the island. The current review provides a broad snapshot of the status of vector-borne infectious diseases and associated risks in Cyprus. Our research has uncovered a pressing issue, the risk of the spread and emergence of various infectious diseases, including West Nile virus and malaria, respectively, due to the presence of *Aedes* and *Anopheles* spp. mosquitoes on the island, while underscoring the animal reservoirs of several pathogenic microorganisms. Our research emphasizes the importance of the One Health approach and the collaboration between communities for the improvement of vector control strategies to limit the spread of vector borne diseases.

## 1. Introduction

The island of Cyprus, located in the eastern part of the Mediterranean Sea, has been prone to infectious diseases throughout history. It has been at the intersection of various civilizations and human/animal migrations, with a warm, subtropical climate. The contagious disease landscape in Cyprus is expected to change as the earth’s climate changes, along with the constant shift in regional demographics. The island of Cyprus is predicted to experience climate change at comparatively higher levels, leading to increased temperatures and extreme weather events [1]. The increased global temperatures coupled with extreme climate events have been known to cause increases in vector-borne infectious diseases, particularly mosquito-borne ones [2].

The island of Cyprus struggled with malaria well into the 1960s until the island was declared malaria-free in 1967 [3]. Most recently, mosquito-borne diseases, such as West Nile virus (WNV) infections, have been causing outbreaks on the island [4]. Pathogens associated with sandflies, such as the protozoan parasite *Leishmania* Ross, 1903 spp., have been impacting animals and humans in Cyprus, where recent reports are pointing toward an increase in human leishmaniasis cases [5]. Furthermore, a rise in the number of imported malaria cases, coupled with the identification of various *Anopheles* Meigen, 1818 mosquito species on the island, is raising concern [6]. In the era of antimicrobial resistance crisis, close attention must not only be paid to prevent the introduction of new infectious agents to the island but also to prevent the spread of drug resistance, as it has been a significant issue globally and in the region [7]. The introduction of invasive mosquito species like *Aedes* Meigen, 1818 spp. could also heavily affect the tourism industry on which the island economically relies [8]. As previously reviewed by Seyer-Cagatan and colleagues, vector-borne diseases are a growing health concern in Cyprus [9].

Many of the vector-borne diseases as well as antimicrobial resistant pathogens are associated with zoonoses, highlighting the importance of the “One Health” approach. This approach underscores the interconnectedness of human, animal, and ecosystem health. The disruption or mishandling of one of these could lead to catastrophic outcomes for the others [10]. While we believe that modeling studies should be validated with field data, these studies, coupled with systematic reporting, to help predict disease and vector spread patterns are crucial to control vector-borne diseases [11].

Currently, the United Nations Development Program (UNDP) has an ongoing multilocation, bicommunal program in Cyprus that aims to trap, identify, and report various invasive mosquito species on the island. Similarly, the European Union (EU), Commission Directorate-General for Health and Food Safety, Health Security Committee, has been supportive of the pilot project on using sterile mosquitoes in *Aedes aegypti* (Linnaeus, 1762) elimination in Cyprus. However, the current landscape regarding the surveillance and reporting of vectors and associated diseases should be collaboratively improved at a multicommunal level with the support of all communities and experts on the island to prevent the spread of disease.

Furthermore, as many of the discussed infections are relatively new for the island of Cyprus, we cannot ignore the possibility of underdiagnosis. Potentially, subclinical human or veterinary infections that remain subclinical are not diagnosed and reported, suggesting that the data identified and reported only reflect a subset of cases. Furthermore, while certain vector-borne infections like WNV, dengue, and malaria are notifiable diseases in the EU, others such as anaplasmosis are not, which, especially when coupled with underdiagnosis, can lead to gaps in disease reporting.

To address the recent increase in vector-borne diseases and highlight the current status of vector-borne diseases in Cyprus, we carried out a narrative review of the relevant literature to gain insight into potential threats and associated precautionary mechanisms. The main aims of our review were to provide a comprehensive outlook toward the snapshot of the vector-borne disease status of Cyprus for the past 20 years, highlight risks, and provide approaches for prevention, as vector/disease spread is a pressing issue. To these ends, we included findings reported throughout the island concerning both veterinary and human cases, predictions, and findings of vector-borne infectious microorganisms. We offer insight into the potential risks associated with emerging diseases and preventive measures.

## 2. Materials and Methods

A literature search was performed using the electronic databases NCBI Pubmed, Scopus, and Google Scholar. This study was carried out in Cyprus regarding vector-transmitted pathogenic viruses, bacteria, and protozoan parasites with a history or potential of impacting the island and its human population. The searched terms included West Nile virus, Dengue virus, *Rickettsia*, *Anaplasma*, *Ehrlichia*, *Coxiella*, *Malaria* or *Plasmodium*, *Leishmania*, *Borrelia*, Crimean Congo Hemorrhagic fever virus, Zika virus, Phlebovirus, Chikungunya virus separately along with “Cyprus” with Boolean operators “OR” and “AND”. As a narrative review style was selected, all articles written in English or with an English abstract were screened, and those reporting findings or predictions associated with humans or animals in Cyprus, with the word “Cyprus” in the title, abstract, or body, were included. All papers published from 2005 to 2025 were included, spanning a 20-year time period by the end of data collection. Exclusion criteria included publications reporting data from different locations of the world and articles solely investigating therapeutic approaches (Figure S1). The results are presented on a pathogen-based manner, starting with viruses, and then bacteria and protozoan parasites, respectively. While this work results from the robust research of 3 databases and demonstrates a comprehensive overview of vector-borne diseases on the island of Cyprus in the past 20 years, there is a possibility that our keywords may not have targeted all the related articles, which may have caused oversight in including all possible articles during the screening process. Furthermore, we acknowledge the reporting of studies relying strictly on seropositivity and recognize that seropositivity does not always indicate past infection unlike seroconversion. Similarly, in certain cases, serologic positivity may be low due to waning antibody levels leading to an underestimation of infections. To this end, there may be over/underestimation of infections strictly relying on seropositivity results.

## 3. Results and Discussion

The literature search results on vector-borne diseases in Cyprus are presented in Table 1 to demonstrate the etiological agents, the reported cases, and prevalence and seropositivity rates. This table highlights the observed prevalence of particular infections, such as West Nile virus and *Leishmania* spp. infections, increasing in seropositivity and prevalence in recent years while demonstrating the historical, consistent presence of vector-borne bacterial pathogens, particularly in animal reservoirs across the island.

**Table 1:** Studies and reports of vector-borne diseases in Cyprus. PCR: polymerase chain reaction; SFSV: sandfly fever Sicilian virus; SNV: Sin Nombre Virus; TOSV: Toscana virus.

Etiological Agent	Prevalence, Seropositivity, Case	Study Year	Reference
Viruses			
Dengue Virus	2 cases of imported dengue cases in Cyprus.	2024	[12]
West Nile Virus	First neuroinvasive human case of WNV infection in Cyprus.	2017	[4]
	Complete genome sequence of the first human neuroinvasive WNV infection, placing it into genetic lineage 1, clade 1a, cluster 2.	2017	[13]
	Seroprevalence rate for anti-WNV IgG of 5%; anti-WNV IgM 17 out of the 127 patients with symptoms.	2019	[14]
	2 (0.3%) IgM+ and 31 (4.1%) IgG+ cases out of 760 sera screened.	2020	[15]
	1.3% seropositivity rate detected out of 836 avian blood samples from 44 migratory and local bird species.	2021	[16]
	WNV RNA detected in *Culex pipiens* mosquitoes from Nicosia (Figure 1) (2019).	2022	[17]
Phleboviruses	SFSV, SNV, and TOSV seropositivity associated with symptomatic disease (2007)	2007	[18]
	Identification of TOSV in sandflies from Northern Cyprus	2014	[19]
	Neutralizing antibodies against TOSV, SFSV, Arbia, and Adana viruses (Salehabad viruses) in dogs.	2016	[20]
	SFSV antibodies detected in a 45-year-old tourist with associated symptoms.	2018	[21]
Zika virus	Absence of seropositivity among blood donors in North Cyprus.	2021	[22]
**Bacteria**			
*Rickettsia* spp.	First detection of *Rickettsia felis* in *Ctenocephalides felis* fleas in rats in Cyprus.	2006	[23]
	A novel, uncultured *Rickettsia* species was identified in ticks in Cyprus (*Rickettsia* species strain Tselenti).	2013	[24]
	Cases until 2017 reviewed by Tsioutis et al. (2017) as follows: 21 pediatric cases in Cyprus from 2000 to 2006 with *R. typhi* infection and a case during pregnancy by Koliou et al. [25], and 193 human cases of *R. typhi* infection Cyprus from 2000 to 2008 by Psaroulaki et al., 2012 [26].	2017	[27]
	Ectoparasites of 161 dogs and 59 cats in Cyprus carried *Rickettsia massiliae*, *Rickettsia conorii*, *Rickettsia felis* (ticks), and *Rickettsia felis, Rickettsia* spp. (fleas).	2022	[28]
	Ricketsial IgG seropositivity of 2% (6 total) when sera from 300 hunters were screened between 2017 and 2018 from Kyrenia and Rizokarpaso (Figure 1).	2022	[29]
*Anaplasma* spp.	2 (4%) dogs positive for *Anaplasma platys* DNA detected in 47 dogs with clinical leishmaniosis and 3 (3%) in 87 healthy control dogs.	2018	[30]
	Anti-*Anaplasma phagocytophilum/Anaplasma platys* antibodies in 5 out of 134 dogs.	2018	[30]
	3 ticks from 161 dogs in Cyprus had *Anaplasma platys.*	2022	[28]
*Ehrlichia* spp.	6 (12%) dogs positive for *Ehrlichia canis* DNA detected in 47 dogs with Clinical leishmaniosis and 1 (1%) in 87 healthy control dogs.	2018	[30]
	*Ehrlichia ewingii* antibodies in 17 out of 134 tested dogs.	2018	[30]
	Increased risk for *E. canis/ E. ewingii* seropositivity in dogs with clinical leishmaniasis compared to healthy dogs.	2018	[30]
*Borrelia* spp.	47 dogs with clinical leishmaniosis were screened for anti-*Borrelia burgdorferii* antibodies, but no seropositivity was detected.	2018	[30]
**Protozoan Parasites**			
*Leishmania* spp.	Patient diagnosed with *L. donovani* complex cutaneous leishmaniasis after a 3-day visit to north Cyprus.	2015	[31]
	Animal and human cases along with seropositivity rates detailed by Schou et al., between 1998 and 2018.	2020	[32]
	Since 2018, 47 dogs were identified with leishmaniosis.	2018	[30]
	1 *P. papatasi* sandfly identified as positive for *L. major* from Cyprus using PCR-based detection methods.	2022	[33]
	*L. infantum* IgG positivity was 4.7% (14/300) in healthy donors from north Cyprus.	2022	[34]
*Plasmodium* spp.	Three cases of vivax malaria in individuals returning to UK from Cyprus were reported in 2017.	2020	[35]
	13 patients were diagnosed with malaria between 2016 and 2019, and *Plasmodium falciparum, Plasmodium vivax*, and *Plasmodium ovale* species were identified, revealing a significant increase in imported cases in 2019.	2021	[22]

**Figure 1:**
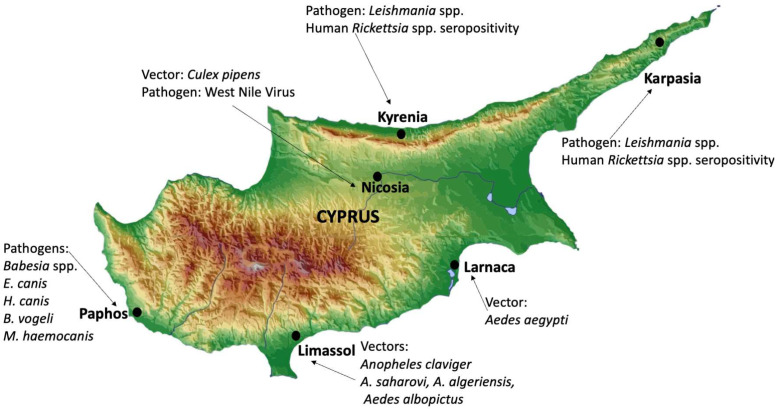
Various vectors and vector-borne pathogens or their seropositivity identified at specific locations on the island of Cyprus.

### 3.1. Viruses

#### 3.1.1. Dengue Virus

Dengue virus (DENV) is notorious for causing the most prevalent arbovirus-associated diseases globally [36]. *Aedes aegypti* is the principal vector for DENV; however, *Ae. albopictus* (Skuse, 1894) is a highly competent vector for its spread as well [37]. According to the World Health Organization (WHO), while some dengue cases may or may not have symptoms or be self-limiting with or without warning signs, others can be classified as Severe Dengue.

DENV is a positive-sense RNA flavivirus with four serotypes in circulation [38]. Dengue virus infection is associated with a concerning immunological phenomenon termed “antibody-dependent enhancement” (ADE), where a prior infection with an unrelated serotype generally leads to a more severe outcome of a future infection. Since a previous infection with a DENV serotype can make any future infections more severe due to ADE, an earlier laboratory-documented infection with a DENV serotype is a requirement to qualify for vaccine uptake [39]. Therefore, DENV outbreaks remain a real threat, particularly in countries with no documented local DENV cases and with *Aedes* mosquito invasion, as in Cyprus.

Various studies highlighted how *Ae. aegypti* and *Ae. albopictus* mosquitoes have been spreading in Europe, finally reaching Cyprus [40], where their spread is predicted to expand. In line with global changes in climate patterns, simulation models project that habitat suitability for *Ae*. *albopictus* could expand to expose 2.4 billion people by 2050 [41]. The hypotheses generated by environmentally guided mathematical models further indicate that *Ae. albopictus* could withstand harsh winter conditions via a diapausing mechanism [42], rendering it more likely to persist throughout the year [43] (Table 2).

One approach used proactively in Cyprus to avoid such an outbreak has been releasing irradiated, sterilized mosquitoes [44]. This pilot project, which was kickstarted in June 2023, is the first one targeted against *Ae. aegypti* in Europe.

Incidentally, two DENV import cases have been reported on the island in November 2023 [12]. Two women returning from Saudi Arabia were diagnosed with dengue fever, and their treatment was carried out successfully on the island. This isolated incident exemplifies the threat associated with a potential virus spread on the island since the primary vector, *Ae. aegypti* and others are now found on the island [40].

While this article focuses on the DENV, mainly because it does not have a universal vaccine that can be indiscriminately applied to the whole population and import cases in Cyprus have been reported, it must be remembered that *Ae. aegypti* is also the mosquito species involved in the spread of yellow fever virus in addition to zika and chikungunya viruses. *Ae. albopictus*, a species introduced to the island, can also transmit chikungunya, yellow fever, West Nile, and zika viruses in addition to DENV. While *Ae. aegypti*, an established vector on the island is the vector for zika virus, a study investigating zika seropositivity among blood donors in Cyprus reported no seroprevalence [22]. Besides *Ae. aegypti* and *Ae. albopictus*, other *Aedes* species, such as *Ae. cretinus* Edwards 1921, which the ECDC has reported to have been restricted to Greece and Türkiye, is also present on the island [45], where *Ae. detritus* (Haliday, 1833) and *Ae. caspius* (Pallas, 1771) are also among the identified *Aedes* species in Cyprus [46].

In addition to traditional prevention methods and newer methods used, such as irradiation, more novel approaches are expected to gain traction to prevent arbovirus-associated infections. For instance, recent studies are indicating a symbiotic bacterium named *Rosenbergiella* Mergaert et al., 2013 spp. YN46 isolated from the gut of *Ae. albopictus* mosquitoes in suppressing flavivirus transmission, which might offer novel avenues of research and intervention to prevent flavivirus-associated diseases [47]. While the exciting potential of novel prevention methods is inspiring, all precautionary mechanisms should be in place until revolutionary new approaches are thoroughly tested and implemented. Once a mosquito species is established on the island, it can lead to the transmission of the pathogens it can harbor, depending on which ones it is exposed to. This underscores the importance of multi-communal screening, monitoring, and control efforts.

**Table 2:** Studies identifying or predicting the spread of *Ae*. *albopictus* and *Ae. aegypti* species in Cyprus, vectors for DENV, chikungunya, zika, and yellow fever viruses.

Study	Method Used	Prediction/Identification
Vasques et al. [40]	Morphological and molecular identification	Identified *Ae. albopictus* and *Ae. aegypti* in Larnaka and Limassol (Figure 1)
Proestos et al. [41] and Georgiades et al. [48]	Machine learning and simulation modeling	Predicted 2.4 billion people exposed to *Ae. albopictus* by 2050 within 20 million km^2^
Erguler et al. [43]	Large-scale environmentally driven mathematical model	Hypothesized the survival of *Ae*. *albopictus* through harsh winters

#### 3.1.2. West Nile Virus

Arguably, the most pressing emerging disease in Cyprus is currently the West Nile virus (WNV). WNV is a neurotrophic flavivirus with the ability to cause paralysis, seizures, cognitive dysfunction, and encephalitis. While 80% of people infected with WNV remain asymptomatic, WNV-associated neuro-invasive disease is thought to affect 1% of infected individuals and particularly impact immunosuppressed patients [49]. The potential impact of WNV on public health and healthcare infrastructures is a serious concern that needs to be addressed.

*Culex* mosquitoes are the primary WNV vectors. Specifically, the *Culex* mosquitoes that feed on birds, like *Culex pipiens* Linnaeus, 1758, *Cx. restuans* Theobald, 1901, *Cx. tarsalis* Coquillett, 1896, *Cx. quinquefasciatus* Say 1823, *Cx. nigripalpus* Theobald, 1901, amd *Cx. stigmatosoma* Dyar, 1907 are principal vectors for WNV, where *Ae. albopictus* is considered a competent bridge vector [50].

Cyprus, with its unique location in the Mediterranean Sea, surrounded by Europe, Africa, and the Middle East, is a crucial point on the migratory route for various migratory birds. Bird migration plays a significant role in WNV epidemiology by creating novel disease foci. This was evidenced by a 1.3% seropositivity rate in 836 avian blood samples screened from 44 different migratory bird species captured in Cyprus [16]. Furthermore, field-collected *Cx. pipiens* mosquitoes from Nicosia (Figure 1), tested positive for WNV [17].

Recent studies further emphasize WNV IgG seropositivity in humans at 4.1%, translating to 31 out of 760 sera samples screened, highlighting the current prevalence of WNV on the island [15]. Previous studies demonstrated that sera obtained and screened between 2013 and 2014 revealed 5% seropositivity for West Nile virus IgG [14], suggesting a consistent prevalence.

The first neuro-invasive WNV infection in Cyprus was reported in 2017 [4], followed by the release of the virus’ genome isolated from the infected individual [13]. Genome analysis revealed that the WNV strain isolated belonged to a genetic lineage with previous outbreak records in Europe and Africa [51]. The isolated strain has strong pathogenicity determinants explaining the observed neuro-invasive presentation and infectivity.

We have also contacted the European Center for Disease Control (ECDC) to obtain information regarding reported cases of West Nile virus infections. The retrieved data indicated that the highest reported cases were observed in 2019, with at least 19 reported cases (Table 3). Any aggregation resulting in less than five cases recorded as <5 for anonymity, suggesting that there were between 0 and 4 reports of West Nile virus infections in 2016, 2018, and 2021 (Table 3). Unsurprisingly, most cases were reported in summer and early fall (Table 3). Likely associated with the same outbreak, two deaths occurred in the summer of 2019 in Cyprus due to the West Nile virus. The October 2023 outbreak of WNV on the north of the island caused three (known) cases and led to the death of an 82-year-old patient [52]. Most recently, an 80-year-old from Nicosia was diagnosed with WNV infection in October 2024 leaving him in critical condition, marking it as the first case of 2024 and highlighting the emergency of the WNV infectious landscape, as well as the necessity of arthropod control measures.

Given the periodic outbreaks of WNV and associated deaths occurring every couple of years in Cyprus, it is of utmost importance to implement strict entomological control measures. Equally important is the need to significantly increase public awareness. This is not just a task for health authorities and policymakers, but a collective responsibility that requires the active participation of public health professionals and the general public to prevent further spread of the infection.

#### 3.1.3. Phleboviruses

Phleboviruses transmitted by phlebotomine sandflies belong to the *Phenuiviridae* family, *Bunyavirales* order, and can be associated with several human pathologies ranging from those causing febrile diseases characterized by “3-day-fever” to those with severe neuroinvasive manifestations [53]. Phlebovirus associated diseases referred to as “sandfly fever” or “pappataci fever” have historically impacted Cyprus where Swedish UN Peacekeeping Troops experienced an outbreak in the 1980s, which was associated with sandfly Sicilian virus (SFSV), sandfly Naples virus (SFNV), and Toscana virus (TOSV) [54]. In the early 2000s, a Cypriot strain of SFSV was associated with an outbreak on the island that brough phleboviruses back on the map. More recent studies from 2007 and 2013–2014 revealed the seroprevalence of SFSV, SFNV, and TOSV on the island [14,18]. The zoonotic potential of phleboviruses is conceivable, as a serology screen of dogs from Cyprus revealed neutralizing antibodies against TOSV, SFSV, Arbia virus, and Adana virus (Salehabad viruses) [20]. The most recent incidence of sandfly fever in Cyprus occurred in a 45-year-old woman who stayed on a mountainous village in north Cyprus for 11 days as a tourist and exhibited symptoms associated with sandfly fever upon her return home. Her bloodwork revealed antibodies against SFSV, which led to a diagnosis of “pappataci fever” [21]. While sandfly fevers are generally self-limiting, some, especially those associated with TOSV can sometimes be associated with more serious pathogenesis such as meningitis or meningoencephalitis, emphasizing the need for healthcare professionals to be on the lookout upon encountering cases associated with febrile illness and aseptic meningitis.

### 3.2. Bacteria

#### 3.2.1. *Rickettsia* spp.

Cyprus hosts a rich flora of endemic and local animals, including mammals, reptiles, bird species, and migratory birds that visit the island periodically, likely introducing novel pathogens and exchanging endemic pathogens, including *Rickettsia* da Rocha-Lima 1916 spp. The genus *Rickettsia* can be divided into two subgroups, spotted fever and typhus, which clinically present with fever, headache, myalgias, and other symptoms. Various species of the spotted fever group are associated with human diseases, such as *R. rickettsii* Brumpt 1922, which causes Rocky Mountain Spotted Fever, and *R. akari* Philip et al., 1952, which can cause rickettsial pox. *Rickettsia prowazekii* da Rocha-Lima 1916 and *R. typhi* Wilder 1919 are species from the typhus group associated with human disease-causing epidemic (louse-borne)/recrudescent/sporadic typhus and endemic (murine) typhus, respectively. Among these human pathogens, *R. typhi* has been identified on the island so far, along with many other *Rickettsia* spp., including *R. aeschlimannii* Beati et al. 1997, *R. Rickettsia sibirica* subsp*. mongolitimonae* Fournier et al. 2005, *R. hoogstraalii* Sekeyová et al. 2010, “*Candidatus* R. barbariae” Fournier et al. 2003, *R. conorii* Brumpt 1932, and *R. felis* Bouyer et al. 2001. In particular, rats have been historically well established in the context of Cyprus as being carriers for various *Rickettsia* spp., such as *R. typhi* and *R. felis* [55], as rats have been reported as being potential indicators for Rickettsial dispersal [56].

The prevalence of various *Rickettsia* species on the island has been demonstrated via numerous studies, underscoring the need for a comprehensive understanding of this issue. A subset of the 368 reported local, endemic, and migratory bird species and their ectoparasites were investigated between 2004 and 2006, revealing a 3% positivity rate for *Rickettsia* spp. as well as *Coxiella burnetii* (Derrick 1939) Philip 1948 positivity, which has also been found in fleas in Cyprus [57,58]. While 3% out of 557 screened samples demonstrate a relatively minimal prevalence for *Rickettsia* spp. and *C. burnetii* positivity, *C. burnetii* is associated with a high number of ruminant abortions [59], and it indicates a risk for the potential spread of these pathogens, considering that farming, outdoor activities, and bird hunting culture are well established on both sides of the island. To this end, a 2022 study conducted between 2017 and 2018 revealed 2% seropositivity for *Rickettsia* IgG antibodies in serum samples from 300 hunters on the northern side of Cyprus [29]. Human seropositivity observed in a relatively small screen is concerning, revealing significant Rickettsial exposure of not only the wildlife but also the human population.

Studies carried out on Cypriot mouflon (*Ovis orientalis ophion* Blyth, 1841), an endemic wild sheep species in Cyprus, revealed 30% positivity for *Rickettsia*, where an even higher percentage was observed in their ectoparasites [60]. While the Cypriot mouflon is considered an endangered species and its hunting is illegal, the detection of *Rickettsia* spp., both in their blood and in their ectoparasites, reveal the presence and circulation of this pathogen in their habitat and likely in other members of the ecological network.

There have been reports of various types of *Rickettsia* species detected in ticks and fleas obtained from cats and dogs in Cyprus, such as *R. massiliae* Beati and Raoult 1993, *R. conorii*, and *R. felis* [28]. This information is crucial, as companion animals such as cats have historically been an important part of Cypriot culture. Stray cats tend to be in close contact with the Cypriot population, and pet ownership is generally expected.

One of the most significant outbreaks of *Rickettsia* infection in Cyprus occurred between 2000 and 2006 when 21 pediatric murine typhus cases caused by *R. typhi* [25] as well as a case of murine typhus in pregnancy [61], a rare occurrence as reviewed by Tsioutis and colleagues. A novel, uncultured strain of *Rickettsia*, belonging to the spotted fever group named *Rickettsia* species strain Tselenti, has been identified in Cyprus where pathogenic implications of this finding remain unclear [24].

When bacterial pathogens are considered, it is crucial to evaluate the potential and existing antimicrobial resistance profiles of tick-borne bacterial pathogens, particularly in the current era of antimicrobial resistance crisis. The inherent resistance of various *Rickettsia* groups to several antibiotics, such as the resistance or reduced susceptibility of spotted fever group *Rickettsia* spp. toward rifampicin [62], as well as the efflux pump genes identified in *R. conorii*, indicate the problem of antimicrobial resistance (AMR) associated with tick-borne bacterial pathogens. Considering the antimicrobial resistance genes in the tick microbiome [63] and the proximity of these bacterial pathogens to the tick microbiome, the risk of vertical or horizontal spread of AMR genes exists. The ‘One Health’ approach, which considers all variables and stakeholders, is crucial in interfering with the spread of resistant pathogens and is directly linked with the significant global challenge of AMR.

#### 3.2.2. Other Members of the Order Rickettsiales: *Anaplasma & Ehrlichia* spp.

The tick-borne pathogens of the genera *Ehrlichia* Moshkovski, 1945 and *Anaplasma* Theiler 1910, including intracellular bacterial species such as *E*. *chaffeensis* Anderson et al. 1991, *E. ewingii* Anderson et al. 1992, and *A*. *phagocytophilum* Dumler et al. 2001, are capable of causing human monocytic/granulocytic ehrlichiosis and human anaplasmosis, respectively. The first human infection associated with *Anaplasma* in Cyprus was caused by a novel strain of *A. phagocytophilum* in 2008 in a 9-year-old girl who was bitten by a tick [64]. This was followed by an infant with Kawasaki disease who concomitantly was infected by an *Anaplasma* spp. [65]. Subsequent studies revealed the significant problem associated with *Anaplasma*, as the random sampling of goats and sheep revealed *Anaplasma* spp. seroprevalence [66], where the strains were later revealed to be *A. ovis* Lestoquard 1924 **[67]**. *Anaplasma* spp. carriage was also reported in endemic and migratory birds as well as their ectoparasites [58].

So far, no recent human infection associated with these pathogens has come out of Cyprus; however, various *Anaplasma* and *Ehrlichia* species were isolated from animals. For instance, multiple *Anaplasma* species, such as *A. platys* Dumler et al. 2001 and *A. phagocytophilum*, were identified in sheep and goats in Cyprus [66]. Similarly, *E. canis* Donatien and Lestoquard 1935 and *A. platys* were found in dogs [68], where in one instance, a co-infection of *E. canis* with other pathogens like *Hepatozoon canis* (James 1905) Wenyon 1926, *Babesia vogeli* Reichenow 1936, and *Mycoplasma haemocanis* Neimark et al. 2001 was identified [69]. The significant correlation observed between dogs with clinical leishmaniasis and *E. canis* is a cause for concern, considering the prevalence of leishmaniasis in dogs in Cyprus [30].

Similarly to the observations in dogs, an *Ehrlichia/Anaplasma* screening identified an *A. platys* species in a cat. The same study identified the flea-borne bacterial pathogen *Bartonella henselae* (Regnery et al. 1992) Brenner et al. 1993, the bacterium that causes cat scratch disease, in 19 cats [70]. The detection of *B. henselae* in cats is particularly concerning, as cats are an essential part of Cypriot culture. Since cats and humans have been in close contact in Cyprus since prehistoric times and cats continue roaming the streets, freely interacting with people, any disease detected in cats should be monitored because of the potential risk of zoonotic transmission. In addition to the prevalence of *A. platys* and *B. henselae* in ticks identified from dogs and cats in Cyprus, various other vector-borne pathogens with veterinary significance, such as *H. felis* (Patton 1908) Garnham 1954*, H. canis* (James 1905) Wenyon 1926, *B. koehlerae* Kordick et al. 1997, and *B. clarridgeiae* Lawson and Collins 1996 were identified together with *Rickettsia* species in dog/cat ticks and fleas [28]. Studies focusing on dogs did not identify *B. burgdorferi* seropositivity in 47 tested dogs that had confirmed clinical leishmaniasis [71]. No *A. phagocytophilum* positivity was detected in any of the tested tick species, while a case study reported acute anaplasmosis in a 27-year-old female with positive serology and PCR results for *Anaplasma* [72]. This highlights the importance of island-wide ectoparasite control and screenings to ensure the successful prevention of human pathogens from colonizing animals and their ectoparasites, in line with the ‘One Health’ approach.

### 3.3. Protozoan Parasites

#### 3.3.1. *Plasmodium* spp.

Malaria has been considered eradicated in Cyprus since 1967 [73], thanks to the early efforts of Mehmed Aziz and colleagues [3]. However, recent evidence indicated an increase in imported cases due to changes in the demographics of the island, which has brought malaria back into the community lens as a potential concern to address [74]. Adding fuel to the fire, a report titled “Return of vivax malaria in Cyprus” reported that three cases of vivax malaria returned from north Cyprus to the UK in 2017, increasing concerns [35]. While no further reports emerged regarding the presence of endemic vivax malaria on the island of Cyprus, the return of endemic malaria to the island would be a significant concern if *Anopheles* mosquitoes are efficiently established on the island in a widespread manner. Previous reports indicated that a competent vector, *Anopheles claviger* (Meigen, 1804), *A. sacharovi* Favre, 1903, and less competent species, *A. algeriensis* Theobald, 1903 and *A. superpictus* Grassi, 1899, are present on the island [75]. A 2022 study investigating a range of mosquito-breeding habitats in Limassol Port, Cyprus, identified three *Anopheles* species in the region, namely, *A. sacharovi*, *A. algeriensis*, and *A. claviger* (Figure 1) [6]. The role that *A. algeriensis* has played as a vector in transmitting *Plasmodium* Marchiafava & Celli, 1885 species has historically been relatively minor. On the other hand, detecting a malaria vector with higher vectorial competence, such as *A. sacharovi*, is of concern. More widespread, multi-communal, and generalized screenings/control for *Anopheles* mosquitoes on the island are of paramount importance, considering that demographic changes widely influenced by tourism, education, and other means can lead to increased imported malaria cases that could create a perfect combination to trigger the return of malaria to the island.

While it is crucial to prevent future malaria outbreaks using infection control measures, public awareness, entomological control, and similar precautions such as the reporting of any new malaria cases on the island are of utmost importance in determining if an actual public health risk exists. This may affect the decision to obtain, keep, or utilize the WHO-approved R21/Matrix-M vaccine to prevent malaria in children. Considering the constant increase in drug-resistant *P. falciparum* Welch 1897, paying attention to prevention methodologies proves to be even more significant. Interestingly, new studies are pointing toward a relationship between antibiotic usage and malaria. The antibiotics that mosquitoes are exposed to through the ingestion of human blood were demonstrated to impact their microbiota composition and lead to increased malaria transmission [76]. Because Cyprus has among the highest antibiotic usage rates in Europe [77], it is essential to consider if high levels of antibiotic usage could contribute to any potential future malaria outbreaks. As drug resistance is on the increase globally, fueled partly by the irresponsible use of antimicrobials, artemisinin partial resistance in Africa is another cause for concern, emphasizing the need to keep malaria under control via vigilant reporting, investigation, and the study of imported or local malaria cases to prevent outbreaks or treatment difficulties.

#### 3.3.2. *Leishmania* spp.

Protozoan parasites of the genus *Leishmania*, spread by sandflies, are among the predominant parasitic infections that cause significant concern in Cyprus [32]. Leishmaniasis, a parasitic neglected tropical disease, has historically affected the Middle East and Southern Mediterranean regions, with recently increased incidence rates in southern European countries [78]. The disease can present as cutaneous, mucocutaneous, or visceral infection forms in humans. The two *Leishmania* species that primarily affect southern Europe are *L. infantum* Nicolle 1908, which usually causes visceral leishmaniasis, and *L. tropica* Wright 1903, which mainly causes cutaneous leishmaniasis. The emergence of *L. donovanii* (Laveran and Mesnil, 1903) Ross, 1903, which has been causing human cases in the region, as well as the recent identification of *L. major* Yakimoff and Schokhor, 1914 in a sandfly captured in Cyprus are concerning developments regarding the endemic infectious disease landscape of the island [33,79]. Cyprus, a southeastern Mediterranean island at the intersection of Africa, the Middle East, and Europe, is at risk for multiple other species of *Leishmania* to become endemic.

One of the first studies investigating *Leishmania* prevalence in Cyprus identified canine leishmaniasis caused by *L. infantum* zymodeme MON-1. This finding was followed by a study that revealed the presence of the same species, along with four dogs, isolated from the *Phlebotomus tobbi* Adler and Theodor, 1930 sandfly. Yet, the authors noted the absence of the classical vectors for *L. infantum* zymodeme MON 1, *P. neglectus* Tonnoir, 1921, and *P*. *syriacus* Adler and Theodor, 1930 and the fact that *P*. *tobbi* is not an anthropophilic species, possibly explaining the low number of human cases. Interestingly, *P*. *tobbi* isolated from the northern part of Cyprus were co-infected with *L. infantum* and the Toscana virus [80], which can be associated with severe meningoencephalitis. Further studies indicated *L. infantum* MON-1 as one of the most prevalent strains found in Cyprus, mainly affecting dogs. Yet, no human seropositivity was observed then, leading to the term “Cyprus Paradox” [81]. One hypothesis concerning this paradox is that the local population of *P. tobbi* does not bite humans [82]. Interestingly, contradicting the lack of this behavioral tendency of *P*. *tobbi*, this sandfly was demonstrated to preferentially feed on humans and cattle in Türkiye and transmit *L. infantum* [83]. In that case, the threat of the spread of *L. infantum-*associated leishmaniasis to people and farm animals at potentially endemic levels can be possible. To this end, reports indicating an increased exposure of humans to *Leishmania* parasites are on the rise [5,84]. This, in line with increased human infections on the island, is leading to a change in the perception of the Cypriot population that leishmaniasis is no longer a solely veterinary problem [5,84,85,86,87]. Studies on the island’s northern side revealed significant seropositivity of *L*. *infantum*, particularly in people whose professions or hobbies included outdoor activities such as farming or hunting [5]. At the same time, the knowledge level about the disease correlated strongly with the level of education [34], once again highlighting the importance of education and public awareness campaigns to interfere with infectious disease outbreaks.

In addition to the prevalence of *L. infantum*, *L*. *donovani* has also been identified on the island and has led to human cases [85]. For instance, one of the first identified human cases in Cyprus was a 9-month-old girl who was co-infected with *L. donovani* and Epstein–Barr virus [88]. This was followed by a travel-associated cutaneous leishmaniasis case, which was diagnosed as *L. donovani/infantum* complex [89]. Later on, a tourist visiting north Cyprus (Lapithos/Lapta, Kyrenia) for only three days was diagnosed with cutaneous leishmaniasis (Figure 1) [31]. Polymerase chain reaction (PCR) confirmed that the pathogen belonged to *L. donovani* complex (*L. donovani* and *L. infantum/chagasi* Cunha and Chagas, 1937), which more commonly causes visceral leishmaniasis [31]. Another study observed that MON-37 strains from Cyprus were different from all other MON-37 strains, suggesting that they can be autochthonous [90]. The various novel strains identified on the island could reflect the changing demographic structure, tourism, and migration, which could impact infectious diseases profiles.

In the current era of antimicrobial resistance crisis, drug resistance associated with *Leishmania* species in the region is a growing concern such that expressed multidrug resistance genes were detected from *L. infantum* and *L. donovani* species isolated from Cyprus [91]. A concerning finding has been that a more significant fraction of canine *L. infantum* isolates from Cyprus had higher antimicrobial efflux rates than countries like Greece. This could be explained by the increased use of antileishmanial drugs in Cyprus, as there have been few human leishmaniasis cases on the island where dog treatment regulations are not strict, emphasizing the significance of a multidisciplinary approach in infectious diseases and the “One Health” concept [91].

Interestingly, dogs are not the only veterinary concern, as *L*. *infantum* was shown to have a 4.4% feline prevalence in Cyprus as well [90,92]. Historically, leishmaniasis has been considered primarily a canine disease by the Cypriot community, as an increase in dogs has been in the public eye in recent years [93,94]. Two studies carried out in Cyprus 20 years apart revealed an increase in canine leishmaniosis seroprevalence; however, as one study was carried out in the north, where the other was carried out in the southern side of the island using different methodologies, it remains to be investigated if the differential results indicate a longitudinal timewise increase in canine leishmaniasis cases or if it is due to the geographical distribution or both [95,96].

Modeling studies suggest a low agreement between *Leishmania* presence and its vector distribution in Cyprus and surrounding areas, which highlight the need for increased reporting and vector surveillance [97]. Effective monitoring and disease reporting can help provide better insight on infection epidemiology in endemic areas and monitor potential spread to non-endemic regions.

Overall, the presence of human leishmaniasis cases, human pathogenic *Leishmania* species on the island, and the looming threat of drug resistance all underline the urgent need for efficient control mechanisms. This underscores the critical importance of the everyday, multi-communal approach to entomological control.

## 4. Conclusions

The present review provides the most current information on vector-borne parasitic, viral and bacterial diseases in Cyprus, a crucial resource for addressing these health threats while highlighting the associated risks.

This research uncovered a pressing issue: the risk of the emergence of infections like malaria due to the presence of various *Anopheles* spp. on the island. Similarly, the establishment of the *Ae. aegypti* vector for chikungunya, yellow fever, zika, and dengue virus on the island clearly indicates the potential risk of these infections if immediate precautions are not taken. However, the absence of chikungunya, yellow fever, and zika infections so far, despite the presence of the vector, is a fortunate situation. The presence of vector-borne diseases of parasite origin, such as leishmaniasis, and viral vector-borne diseases, such as West Nile virus, were identified in the present research. Specific vector-borne bacterial infectious diseases mainly caused by *Rickettsia* species have also been present on the island. Animal reservoirs of various pathogenic *Rickettsia* spp., as well as *Leishmania* spp., were shown to be present as well. Animal carriers of *Ehrlichia* and *Anaplasma* spp. were observed, whereas human infection cases have been rare; however, reporting bias cannot be excluded. Despite two imported cases, dengue is not considered an immediate threat, but strict monitoring and screening practices should be in place, considering the expansion of its vectors and their associated risk.

It is of utmost importance to implement precautionary arthropod control measures in line with the “One Health” concept. United efforts in monitoring and prevention are crucial to avert potential outbreaks and public health concerns. Only through collaboration will the effective control of these health threats be possible. For instance, in order to keep vector populations and diversity in check, the regular monitoring of vector populations, such as mosquitoes and ticks, should be collaboratively carried out in order to identify species and assess population densities. To achieve this goal, geographic information systems (GISs) can be utilized to map vector habitats and track changes over time, where this can be coupled with modeling studies.

As it was carried out on the island in the 1940s, leading to the eradication of malaria form Cyprus, environmental management strategies such as the elimination of stagnant water sources, breeding grounds for mosquitos, should be efficiently implemented. Larvivorous fish populations should be maintained at effective levels and introduced to standing water bodies as a means of larval control. Similarly, preserving the ecosystem, namely, protecting and encouraging the breeding of natural vector predators such as birds and amphibians, can be increased as a biological control mechanism. This type of biological control strategy can be supplemented with chemical control approaches, in which insecticides and larvicides are judiciously applied to minimize impact on humans and the environment. To avoid the development of insecticide resistance in the insect vectors, different classes of insecticides should be introduced in a rotating manner. As previously proposed, the utilization of programs like irradiated mosquitoes could be promising strategies to limit invasive species.

Collaboration and communication with the community and general public is of utmost importance to prevent the spread of vector-borne diseases. Public awareness regarding vector-borne diseases on the island should be increased to ensure the public’s compliance with the prevention measures needed to achieve a healthier island. Public awareness campaigns can be improved to educate communities about vector-borne diseases and prevention measures such as removing stagnant water sources and using personal protection/insect repellent. This can be achieved by partnering with schools and community organizations and involving healthcare providers to ensure proper patient education and disease reporting.

Several funding bodies including the EU and UNDP are supporting initiatives to track vectors on the island. Allocating increased budgets for disease prevention and research programs while recruiting further international support can also help ensure that the island avoids potential crises associated with vector borne diseases.

To maximize the efficacy of these approaches, they should be implemented in a coordinated and integrated way. Regular evaluation and necessary modifications should be applied in order to address shifting environmental conditions and vector behaviors.

Overall, this work calls for mandatory reporting protocols, a European-level monitoring of vector habitats, and screenings for animal hosts and their ectoparasites. It is crucial to approach this, recognizing the intimate interlinkages between the environment, animal health, and human health.

## Figures and Tables

**Table 3:** Number of reported cases of West Nile virus infection from Cyprus based on The European Surveillance System, by year and month *.

Year	Month	Place of Infection	Number of Cases **
2016	8	Cyprus	<5
2018	9	Cyprus	<5
2019	7	Cyprus	<5
2019	8	Cyprus	<5
2019	8	Cyprus	14
2019	9	Cyprus	5
2019	10	Cyprus	<5
2021	7	Cyprus	<5

* Data from The European Surveillance System (TESSy), provided by the WHO and Ministries of Health and released by the ECDC; ** any aggregation resulting in less than five cases has been recorded as <5 for anonymity.

## Data Availability

No new data were created or analyzed in this study.

## Supplementary Materials

The following supporting information can be downloaded at: https://www.mdpi.com/article/10.3390/microorganisms13040726/s1, Figure S1: PRISMA flow chart of data identification and selection.

## References

[1] Lange M.A. (2019). Impacts of Climate Change on the Eastern Mediterranean and the Middle East and North Africa Region and the Water–Energy Nexus. Atmosphere.

[2] Pavia G., Branda F., Ciccozzi A., Romano C., Locci C., Azzena I., Pascale N., Marascio N., Quirino A., Gigliotti S. (2025). The issue of climate change and the spread of tropical diseases in Europe and Italy: Vector biology, disease transmission, genome-based monitoring and public health implications. Infect. Dis..

[3] Shelley H. (1949). Mehmed Aziz *Anopheles* eradication in Cyprus. Br. Med. J..

[4] Paphitou N.I., Tourvas A., Floridou D., Richter J., Tryfonos C., Christodoulou C. (2017). The first human case of neuroinvasive West Nile virus infection identified in Cyprus. J. Infect. Public Health.

[5] Ruh E., Bostanci A., Kunter V., Tosun O., Imir T., Schallig H., Taylan-Ozkan A. (2017). Leishmaniasis in northern Cyprus: Human cases and their association with risk factors. J. Vector Borne Dis..

[6] Martinou A.F., Athanasiou K., Shawcross K. (2022). Monitoring for Native and Invasive Mosquitoes at the Limassol Port in Cyprus. Med. Sci. Forum.

[7] Adam M., Nahzat S., Kakar Q., Assada M., Witkowski B., Tag Eldin Elshafie A., Abuobaida D., Safi N., Khan M.A., Nagi M. (2023). Antimalarial drug efficacy and resistance in malaria-endemic countries in HANMAT-PIAM_net countries of the Eastern Mediterranean Region 2016-2020: Clinical and genetic studies. Trop. Med. Int. Health.

[8] Piccinno R., Fiorenza G., Vasquez M.I., Bouyer J., Notarides G., Gomulski L.M., Meletiou S., Akiner M., Michaelakis A., Forneris F. (2025). On the tracks of an uninvited guest, the Asian tiger mosquito, *Aedes albopictus* in Cyprus. Parasites Vectors.

[9] Seyer-Cagatan A., Ruh E., Taylan-Ozkan A. (2024). Vector-borne diseases in Cyprus: A detailed review of the literature. Trop. Biomed..

[10] Abreu F.V.S.d., de Andreazzi C.S., Neves M.S.A.S., Meneguete P.S., Ribeiro M.S., Dias C.M.G., de Albuquerque Motta M., Barcellos C., Romão A.R., Magalhães M.d.A.F.M. (2022). Ecological and environmental factors affecting transmission of sylvatic yellow fever in the 2017-2019 outbreak in the Atlantic Forest, Brazil. Parasites Vectors.

[11] Kiryluk H.D., Beard C.B., Holcomb K.M. (2024). The use of environmental data in descriptive and predictive models of vector-borne disease in North America. J. Med. Entomol..

[12] Shkurko J. (2023). Women Hospitalised with Dengue Fever ‘in Good Conditions’. Cyprus Mail.

[13] Richter J., Tryfonos C., Tourvas A., Floridou D., Paphitou N.I., Christodoulou C. (2017). Complete Genome Sequence of West Nile Virus (WNV) from the First Human Case of Neuroinvasive WNV Infection in Cyprus. Genome Announc..

[14] Billioud G., Tryfonos C., Richter J. (2019). The Prevalence of Antibodies against Sandfly Fever Viruses and West Nile Virus in Cyprus. J. Arthropod Borne Dis..

[15] Balaman N., Gazi U., Imir T., Sanlidag T., Ruh E., Tosun O., Ozkul A., Taylan-Ozkan A. (2020). Serological screening of West Nile virus among blood donors in northern Cyprus. J. Med. Virol..

[16] Pallari C.T., Efstathiou A., Moysi M., Papanikolas N., Christodoulou V., Mazeris A., Koliou M., Kirschel A.N.G. (2021). Evidence of West Nile virus seropositivity in wild birds on the island of Cyprus. Comp. Immunol. Microbiol. Infect. Dis..

[17] Pallari C.T., Christodoulou V., Koliou M., Kirschel A.N.G. (2022). First detection of WNV RNA presence in field-collected mosquitoes in Cyprus. Acta Trop..

[18] Konstantinou G.N., Papa A., Antoniadis A. (2007). Sandfly-fever outbreak in Cyprus: Are phleboviruses still a health problem?. Travel. Med. Infect. Dis..

[19] Ergunay K., Gunay F., Erisoz Kasap O., Oter K., Gargari S., Karaoglu T., Tezcan S., Cabalar M., Yildirim Y., Emekdas G. (2014). Serological, molecular and entomological surveillance demonstrates widespread circulation of West Nile virus in Turkey. PLoS Negl. Trop. Dis..

[20] Alwassouf S., Christodoulou V., Bichaud L., Ntais P., Mazeris A., Antoniou M., Charrel R.N. (2016). Seroprevalence of Sandfly-Borne Phleboviruses Belonging to Three Serocomplexes (Sandfly fever Naples, Sandfly fever Sicilian and Salehabad) in Dogs from Greece and Cyprus Using Neutralization Test. PLoS Negl. Trop. Dis..

[21] Stahn B., Sudeck H., Frickmann H., Krüger A., Burchard H.G., Wiemer D. (2018). Sandfly fever-a “neglected” disease. Hautarzt.

[22] Abushoufa F., Arikan A., Sanlidag T., Guvenir M., Guler E., Suer K. (2021). Absence of Zika Virus Seroprevalence Among Blood Donors in Northern Cyprus. J. Infect. Dev. Ctries..

[23] Psaroulaki A., Antoniou M., Papaeustathiou A., Toumazos P., Loukaides F., Tselentis Y. (2006). First detection of *Rickettsia felis* in *Ctenocephalides felis* fleas parasitizing rats in Cyprus. Am. J. Trop. Med. Hyg..

[24] Sandalakis V., Chochlakis D., Ioannou I., Psaroulaki A. (2013). Identification of a novel uncultured Rickettsia species strain (*Rickettsia* species strain *Tselenti*) in Cyprus. Am. J. Trop. Med. Hyg..

[25] Koliou M., Psaroulaki A., Georgiou C., Ioannou I., Tselentis Y., Gikas A. (2007). Murine typhus in Cyprus: 21 paediatric cases. Eur. J. Clin. Microbiol. Infect. Dis..

[26] Psaroulaki A., Christou C., Chochlakis D., Tsiligianni I., Sandalakis V., Georgalis L., Ioannou I., Giorgalas G., Tselentis Y. (2012). Murine typhus in Cyprus: A 9-year survey. Trans. R. Soc. Trop. Med. Hyg..

[27] Tsioutis C., Zafeiri M., Avramopoulos A., Prousali E., Miligkos M., Karageorgos S.A. (2017). Clinical and laboratory characteristics, epidemiology, and outcomes of murine typhus: A systematic review. Acta Trop..

[28] Diakou A., Sofroniou D., Paoletti B., Tamvakis A., Kolencik S., Dimzas D., Morelli S., Grillini M., Traversa D. (2022). Ticks, Fleas, and Harboured Pathogens from Dogs and Cats in Cyprus. Pathogens.

[29] Ruh E., Aras S., Gazi U., Celebi B., Tosun O., Sanlidag T., Imir T., Taylan-Ozkan A. (2022). Seroprevalence of rickettsial infection in northern Cyprus: A study among hunters. Trop. Biomed..

[30] Attipa C., Solano-Gallego L., Papasouliotis K., Soutter F., Morris D., Helps C., Carver S., Tasker S. (2018). Association between canine leishmaniosis and *Ehrlichia canis* co-infection: A prospective case-control study. Parasit. Vectors.

[31] de Silva T.I., Debroy Kidambi A., Green S.T., Mahadeva U., Mcgregor A.C., Levy M., Hardcastle N. (2015). Cutaneous leishmaniasis acquired during a brief visit to Cyprus. J. Infect..

[32] Schou C., Filippova M., Quattrocchi A., Karanis P. (2020). The Current Status of Protozoan Parasitic Diseases in Cyprus: A Narrative Literature Review. Environ. Sci. Proc..

[33] Yetişmiş K., Mert U., Caner A., Nalçaci M., Töz S., Özbel Y. (2022). Blood Meal Analysis and Molecular Detection of *Leishmania* DNA in Wild-Caught Sand Flies in Leishmaniasis Endemic Areas of Turkey and Northern Cyprus. Acta Parasitol..

[34] Özdoğaç M., Güler E., Güvenir M., Hürdoğanoğlu U., Kiraz A., Süer K. (2022). Investigation of *Leishmania infantum* Seroprevalance and Leishmaniasis Knowledge Level in Northern Cyprus. Mikrobiyol. Bul..

[35] Emms H., Lee R., Thomas A., Doerholt K., Le Doare K. (2020). Return of vivax malaria in Cyprus. Arch. Dis. Child..

[36] Wilder-Smith A., Hombach J., Ferguson N., Selgelid M., O’Brien K., Vannice K., Barrett A., Ferdinand E., Flasche S., Guzman M. (2019). Deliberations of the Strategic Advisory Group of Experts on Immunization on the use of CYD-TDV dengue vaccine. Lancet Infect. Dis..

[37] Garcia-Rejon J.E., Navarro J., Cigarroa-Toledo N., Baak-Baak C.M. (2021). An Updated Review of the Invasive *Aedes albopictus* in the Americas; Geographical Distribution, Host Feeding Patterns, Arbovirus Infection, and the Potential for Vertical Transmission of Dengue Virus. Insects.

[38] Chen R.E., Smith B.K., Errico J.M., Gordon D.N., Winkler E.S., VanBlargan L.A., Desai C., Handley S.A., Dowd K.A., Amaro-Carambot E. (2021). Implications of a highly divergent dengue virus strain for cross-neutralization, protection, and vaccine immunity. Cell Host Microbe.

[39] Rodríguez-Barraquer I., Salje H., Cummings D.A. (2019). Dengue pre-vaccination screening and positive predictive values. Lancet Infect. Dis..

[40] Vasquez M.I., Notarides G., Meletiou S., Patsoula E., Kavran M., Michaelakis A., Bellini R., Toumazi T., Bouyer J., Petrić D. (2023). Two invasions at once: Update on the introduction of the invasive species *Aedes aegypti* and *Aedes albopictus* in Cyprus—A call for action in Europe. Parasite.

[41] Proestos Y., Christophides G.K., Ergüler K., Tanarhte M., Waldock J., Lelieveld J. (2015). Present and future projections of habitat suitability of the Asian tiger mosquito, a vector of viral pathogens, from global climate simulation. Philos. Trans. R. Soc. Lond. B Biol. Sci..

[42] Tippelt L., Werner D., Kampen H. (2020). Low temperature tolerance of three *Aedes albopictus* strains (Diptera: Culicidae) under constant and fluctuating temperature scenarios. Parasite Vectors.

[43] Erguler K., Smith-Unna S.E., Waldock J., Proestos Y., Christophides G.K., Lelieveld J., Parham P.E. (2016). Large-Scale Modelling of the Environmentally-Driven Population Dynamics of Temperate *Aedes albopictus* (Skuse). PLoS ONE.

[44] Nuclear Technique Used in Europe for First time to Battle Yellow Fever Mosquito Found in Cyprus. https://www.iaea.org/newscenter/pressreleases/nuclear-technique-used-in-europe-for-first-time-to-battle-yellow-fever-mosquito-found-in-cyprus.

[45] Martinou A.F., Fawcett J., Georgiou M., Angelidou I., Philippou M., Schaffner F. (2021). Occurrence of *Aedes cretinus* in Cyprus based on information collected by citizen scientists. J. Eur. Mosq. Control Assoc..

[46] Drakou K., Nikolaou T., Vasquez M., Petric D., Michaelakis A., Kapranas A., Papatheodoulou A., Koliou M. (2020). The Effect of Weather Variables on Mosquito Activity: A Snapshot of the Main Point of Entry of Cyprus. Int. J. Environ. Res. Public Health.

[47] Zhang L., Wang D., Shi P., Li J., Niu J., Chen J., Wang G., Wu L., Chen L., Yang Z. (2024). A naturally isolated symbiotic bacterium suppresses flavivirus transmission by *Aedes* mosquitoes. Science.

[48] Georgiades P., Proestos Y., Lelieveld J., Erguler K. (2023). Machine Learning Modeling of *Aedes albopictus* Habitat Suitability in the 21st Century. Insects.

[49] Mbonde A.A., Gritsch D., Harahsheh E.Y., Kasule S.N., Hasan S., Parsons A.M., Zhang N., Butterfield R., Shiue H., Norville K.A. (2024). Neuroinvasive West Nile Virus Infection in Immunosuppressed and Immunocompetent Adults. JAMA Netw Open.

[50] Bohers C., Vazeille M., Bernaoui L., Pascalin L., Meignan K., Mousson L., Jakerian G., Karch A., de Lamballerie X., Failloux A. (2024). *Aedes albopictus* is a competent vector of five arboviruses affecting human health, greater Paris, France, 2023. Euro Surveill.

[51] Mencattelli G., Ndione M.H.D., Silverj A., Diagne M.M., Curini V., Teodori L., Di Domenico M., Mbaye R., Leone A., Marcacci M. (2023). Spatial and temporal dynamics of West Nile virus between Africa and Europe. Nat. Commun..

[52] (2023). Yeniduzen Batı Nil Virüsü Nedeniyle 1 Kişi Hayatını Kaybetti. https://www.yeniduzen.com/bati-nil-virusu-nedeniyle-1-kisi-hayatini-kaybetti-166942h.htm.

[53] Moriconi M., Rugna G., Calzolari M., Bellini R., Albieri A., Angelini P., Cagarelli R., Landini M.P., Charrel R.N., Varani S. (2017). Phlebotomine sand fly-borne pathogens in the Mediterranean Basin: Human leishmaniasis and phlebovirus infections. PLoS Negl. Trop. Dis..

[54] Eitrem R., Vene S., Niklasson B. (1990). Incidence of sand fly fever among Swedish United Nations soldiers on Cyprus during 1985. Am. J. Trop. Med. Hyg..

[55] Christou C., Psaroulaki A., Antoniou M., Toumazos P., Ioannou I., Mazeris A., Chochlakis D., Tselentis Y. (2010). Rickettsia typhi and *Rickettsia felis* in *Xenopsylla cheopis* and *Leptopsylla segnis* parasitizing rats in Cyprus. Am. J. Trop. Med. Hyg..

[56] Psaroulaki A., Antoniou M., Toumazos P., Mazeris A., Ioannou I., Chochlakis D., Christophi N., Loukaides P., Patsias A., Moschandrea I. (2010). Rats as indicators of the presence and dispersal of six zoonotic microbial agents in Cyprus, an island ecosystem: A seroepidemiological study. Trans. R Soc. Trop. Med. Hyg..

[57] Psaroulaki A., Chochlakis D., Ioannou I., Angelakis E., Tselentis Y. (2014). Presence of *Coxiella burnetii* in fleas in Cyprus. Vector Borne Zoonotic Dis..

[58] Ioannou I., Chochlakis D., Kasinis N., Anayiotos P., Lyssandrou A., Papadopoulos B., Tselentis Y., Psaroulaki A. (2009). Carriage of *Rickettsia* spp., *Coxiella burnetii* and *Anaplasma* spp. by endemic and migratory wild birds and their ectoparasites in Cyprus. Clin. Microbiol. Infect.

[59] Cantas L., Muwonge A., Sareyyupoglu B., Yardimci H., Skjerve E. (2011). Q fever abortions in ruminants and associated on-farm risk factors in northern Cyprus. BMC Vet. Res..

[60] Ioannou I., Sandalakis V., Kassinis N., Chochlakis D., Papadopoulos B., Loukaides F., Tselentis Y., Psaroulaki A. (2011). Tick-borne bacteria in mouflons and their ectoparasites in Cyprus. J. Wildl. Dis..

[61] Koliou M., Christoforou C., Soteriades E.S. (2007). Murine typhus in pregnancy: A case report from Cyprus. Scand. J. Infect. Dis..

[62] Amoros J., Fattar N., Buysse M., Louni M., Bertaux J., Bouchon D., Duron O. (2024). Reassessment of the genetic basis of natural rifampin resistance in the genus Rickettsia. Microbiologyopen.

[63] Chigwada A.D., Mapholi N.O., Ogola H.J.O., Mbizeni S., Masebe T.M. (2022). Pathogenic and Endosymbiotic Bacteria and Their Associated Antibiotic Resistance Biomarkers in Amblyomma and Hyalomma Ticks Infesting Nguni Cattle (*Bos* spp.). Pathogens.

[64] Psaroulaki A., Koliou M., Chochlakis D., Ioannou I., Mazeri S., Tselentis Y. (2008). *Anaplasma phagocytophilum* infection in a child. Pediatr. Infect. Dis. J..

[65] Chochlakis D., Koliou M., Ioannou I., Tselentis Y., Psaroulaki A. (2009). Kawasaki disease and *Anaplasma* sp. infection of an infant in Cyprus. Int. J. Infect. Dis..

[66] Chochlakis D., Ioannou I., Sharif L., Kokkini S., Hristophi N., Dimitriou T., Tselentis Y., Psaroulaki A. (2009). Prevalence of *Anaplasma* sp. in goats and sheep in Cyprus. Vector Borne Zoonotic. Dis..

[67] Psaroulaki A., Chochlakis D., Sandalakis V., Vranakis I., Ioannou I., Tselentis Y. (2009). Phylogentic analysis of *Anaplasma ovis* strains isolated from sheep and goats using groEL and mps4 genes. Vet. Microbiol..

[68] Bouzouraa T., René-Martellet M., Chêne J., Attipa C., Lebert I., Chalvet-Monfray K., Cadoré J., Halos L., Chabanne L. (2016). Clinical and laboratory features of canine *Anaplasma platys* infection in 32 naturally infected dogs in the Mediterranean basin. Ticks Tick Borne Dis..

[69] Attipa C., Hicks C.A.E., Barker E.N., Christodoulou V., Neofytou K., Mylonakis M.E., Siarkou V.I., Vingopoulou E.I., Soutter F., Chochlakis D. (2017). Canine tick-borne pathogens in Cyprus and a unique canine case of multiple co-infections. Ticks Tick Borne Dis..

[70] Angelakis E., Raoult D. (2014). Pathogenicity and treatment of Bartonella infections. Int. J. Antimicrob. Agents.

[71] Attipa C., Solano-Gallego L., Leutenegger C.M., Papasouliotis K., Soutter F., Balzer J., Carver S., Buch J.S., Tasker S. (2019). Associations between clinical canine leishmaniosis and multiple vector-borne co-infections: A case-control serological study. BMC Vet. Res..

[72] Psaroulaki A., Chochlakis D., Ioannou I., Florentia A., Gikas A., Tselentis Y. (2009). Acute anaplasmosis in humans in Cyprus. Clin. Microbiol. Infect..

[73] Constantinou K. (1998). *Anopheles* (malaria) eradication in Cyprus. Parassitologia.

[74] Güler E., Özbilgin A., Çavuş İ., Şanlıdağ T., Süer K. (2020). Evaluation of Imported Malaria Cases in Northern Cyprus between 2016 and 2019: First Data Series. Turkiye Parazitol. Derg..

[75] Violaris M., Vasquez M.I., Samanidou A., Wirth M.C., Hadjivassilis A. (2009). The mosquito fauna of the Republic of Cyprus: A revised list. J. Am. Mosq. Control Assoc..

[76] Gendrin M., Rodgers F.H., Yerbanga R.S., Ouédraogo J.B., Basáñez M., Cohuet A., Christophides G.K. (2015). Antibiotics in ingested human blood affect the mosquito microbiota and capacity to transmit malaria. Nat. Commun..

[77] European Centre for Disease Prevention and Control (ECDC) (2022). Antimicrobial Resistance in the EU/EEA—A One Health Response.

[78] Todeschini R., Musti M.A., Pandolfi P., Troncatti M., Baldini M., Resi D., Natalini S., Bergamini F., Galletti G., Santi A. (2024). Re-emergence of human leishmaniasis in northern Italy, 2004 to 2022: A retrospective analysis. Euro. Surveill..

[79] Yetismis K., Erguler K., Angelidou I., Yetismis S., Fawcett J., Foroma E., Jarraud N., Ozbel Y., Martinou A.F. (2022). Establishing the *Aedes* watch out network, the first island-wide mosquito citizen-science initiative in Cyprus within the framework of the Mosquitoes Without Borders project. MBI.

[80] Ergunay K., Kasap O.E., Orsten S., Oter K., Gunay F., Yoldar A.Z.A., Dincer E., Alten B., Ozkul A. (2014). Phlebovirus and *Leishmania* detection in sandflies from eastern Thrace and northern Cyprus. Parasit. Vectors.

[81] Mazeris A., Soteriadou K., Dedet J.P., Haralambous C., Tsatsaris A., Moschandreas J., Messaritakis I., Christodoulou V., Papadopoulos B., Ivovic V. (2010). Leishmaniases and the Cyprus paradox. Am. J. Trop. Med. Hyg..

[82] Léger N., Depaquit J. (2008). *Leishmania donovani* leishmaniasis in Cyprus. Lancet Infect Dis.

[83] Svobodová M., Alten B., Zídková L., Dvorák V., Hlavacková J., Mysková J., Seblová V., Kasap O.E., Belen A., Votýpka J. (2009). Cutaneous leishmaniasis caused by *Leishmania infantum* transmitted by *Phlebotomus tobbi*. Int. J. Parasitol..

[84] Sayili A., Ozkan A.T., Schallig H.D.F.H. (2016). Pediatric Visceral Leishmaniasis Caused by *Leishmania infantum* in Northern Cyprus. Am. J. Trop. Med. Hyg..

[85] Antoniou M., Haralambous C., Mazeris A., Pratlong F., Dedet J., Soteriadou K. (2009). *Leishmania donovani* leishmaniasis in Cyprus. Lancet Infect. Dis..

[86] Koliou M.G., Antoniou Y., Antoniou M., Christodoulou V., Mazeris A., Soteriades E.S. (2014). A cluster of four cases of cutaneous leishmaniasis by *Leishmania donovani* in Cyprus: A case series. J. Med. Case Rep..

[87] Pagliano P., Ascione T. (2017). Pediatric Visceral Leishmaniasis Caused by *Leishmania infantum* in Northern Cyprus. Am. J. Trop. Med. Hyg..

[88] Koliou M.G., Soteriades E.S., Ephros M., Mazeris A., Antoniou M., Elia A., Novelli V. (2008). Hemophagocytic lymphohistiocytosis associated with Epstein Barr virus and *Leishmania donovani* coinfection in a child from Cyprus. J. Pediatr. Hematol. Oncol..

[89] Poeppl W., Walochnik J., Pustelnik T., Auer H., Mooseder G. (2011). Cutaneous leishmaniasis after travel to Cyprus and successful treatment with miltefosine. Am. J. Trop. Med. Hyg..

[90] Alam M.Z., Haralambous C., Kuhls K., Gouzelou E., Sgouras D., Soteriadou K., Schnur L., Pratlong F., Schönian G. (2009). The paraphyletic composition of *Leishmania donovani* zymodeme MON-37 revealed by multilocus microsatellite typing. Microbes Infect..

[91] Tsirigotakis N., Christodoulou V., Ntais P., Mazeris A., Koutala E., Messaritakis I., Antoniou M. (2016). Geographical Distribution of MDR1 Expression in Leishmania Isolates, from Greece and Cyprus, Measured by the Rhodamine-123 Efflux Potential of the Isolates, Using Flow Cytometry. Am. J. Trop. Med. Hyg..

[92] Attipa C., Papasouliotis K., Solano-Gallego L., Baneth G., Nachum-Biala Y., Sarvani E., Knowles T.G., Mengi S., Morris D., Helps C. (2017). Prevalence study and risk factor analysis of selected bacterial, protozoal and viral, including vector-borne, pathogens in cats from Cyprus. Parasit. Vectors.

[93] Beyhan Y.E., Çelebi B., Ergene O., Mungan M. (2016). Seroprevalance of Leishmaniasis in Dogs from Hatay and Burdur Provinces of Turkey and Northern Cyprus. Turkiye Parazitol. Derg..

[94] Töz S.O., Ertabaklar H., Göçmen B., Demir S., Karakuş M., Arserim S.K., Balcıoğlu I.C., Canakçı T., Ozbel Y. (2013). An epidemiological study on canine leishmaniasis (CanL) and sand flies in Northern Cyprus. Turkiye Parazitol. Derg..

[95] Çanakçı T., Kurtdede A., Paşa S., Töz Özensoy S., Özbel Y. (2016). Seroprevalence of Canine Leishmaniasis in Northern Cyprus. Turkiye Parazitol. Derg..

[96] Deplazes P., Grimm F., Papaprodromou M., Cavaliero T., Gramiccia M., Christofi G., Christofi N., Economides P., Eckert J. (1998). Canine leishmaniosis in Cyprus due to *Leishmania infantum* MON 1. Acta Trop..

[97] Berriatua E., Pérez-Cutillas P., Vidal A.G., Briët O.J.T. (2024). The spatial relationship between leishmaniases and sand flies in Europe and neighboring countries. Parasites Vectors.

