# Supplementary figures and images for: Current Risks and Prevention Strategies Against Vector-Borne Diseases in Cyprus
Source: Microorganisms. 2025 Mar 24;13(4):726. doi: 10.3390/microorganisms13040726 (PMC12029801; doi:10.3390/microorganisms13040726)

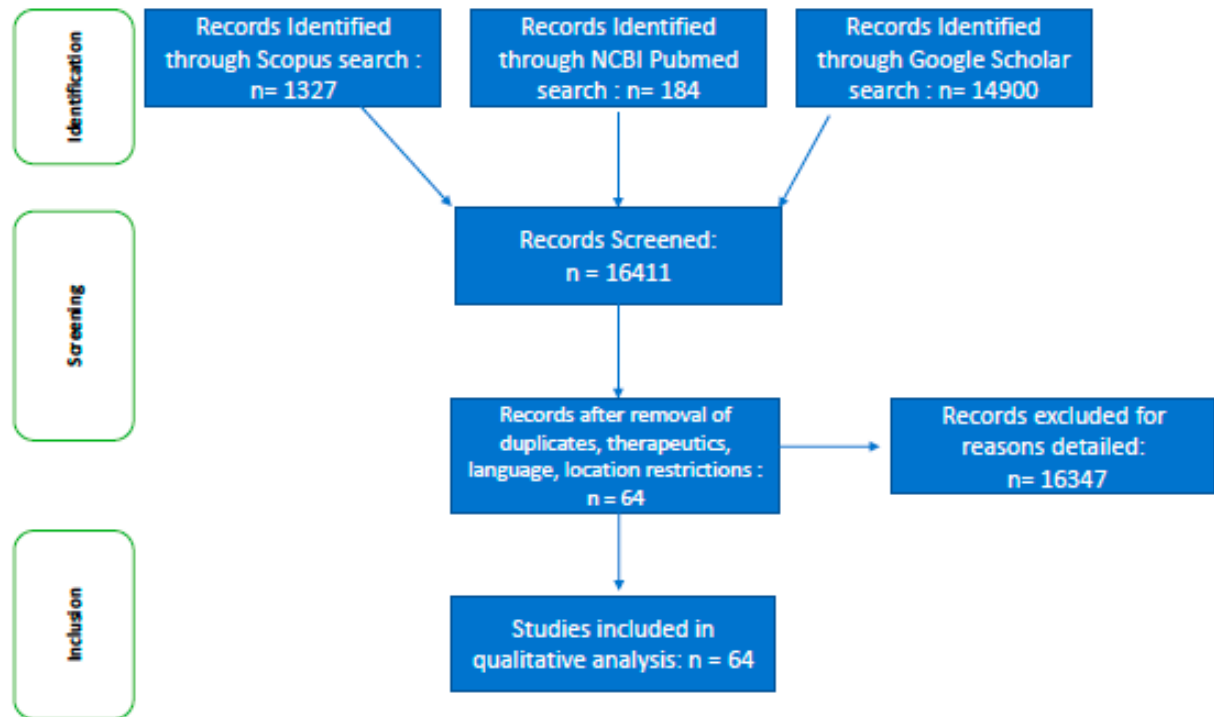

Supplemental Figure S1: PRISMA flow chart of data identification and selection.

Supplement: Supplementary file 1 [file microorganisms-13-00726-s001.zip › microorganisms-3507905-supplementary.pdf]
